# Fibroblast growth factor receptor signaling in cardiomyocytes is protective in the acute phase following ischemia-reperfusion injury

**DOI:** 10.3389/fcvm.2022.1011167

**Published:** 2022-09-23

**Authors:** Dzmitry Matsiukevich, Stacey L. House, Carla Weinheimer, Attila Kovacs, David M. Ornitz

**Affiliations:** ^1^Department of Pediatrics, Washington University in St. Louis School of Medicine, St. Louis, MO, United States; ^2^Department of Developmental Biology, Washington University in St. Louis School of Medicine, St. Louis, MO, United States; ^3^Department of Emergency Medicine, Washington University in St. Louis School of Medicine, St. Louis, MO, United States; ^4^Department of Medicine, Washington University in St. Louis School of Medicine, St. Louis, MO, United States

**Keywords:** fibroblast growth factor receptor, FGFR1, FGFR2, cardiomyocyte, ischemia-reperfusion injury, cardioprotection

## Abstract

Fibroblast growth factor receptors (FGFRs) are expressed in multiple cell types in the adult heart. Previous studies have shown a cardioprotective effect of some FGF ligands in cardiac ischemia-reperfusion (I/R) injury and a protective role for endothelial FGFRs in post-ischemic vascular remodeling. To determine the direct role FGFR signaling in cardiomyocytes in acute cardiac I/R injury, we inactivated *Fgfr1* and *Fgfr2* (CM-DCKO) or activated FGFR1 (CM-caFGFR1) in cardiomyocytes in adult mice prior to I/R injury. In the absence of injury, inactivation of *Fgfr1* and *Fgfr2* in adult cardiomyocytes had no effect on cardiac morphometry or function. When subjected to I/R injury, compared to controls, CM-DCKO mice had significantly increased myocyte death 1 day after reperfusion, and increased infarct size, cardiac dysfunction, and myocyte hypertrophy 7 days after reperfusion. No genotype-dependent effect was observed on post-ischemic cardiomyocyte cross-sectional area and vessel density in areas remote to the infarct. By contrast, transient activation of FGFR1 signaling in cardiomyocytes just prior to the onset of ischemia did not affect outcomes after cardiac I/R injury at 1 day and 7 days after reperfusion. These data demonstrate that endogenous cell-autonomous cardiomyocyte FGFR signaling supports the survival of cardiomyocytes in the acute phase following cardiac I/R injury and that this cardioprotection results in continued improved outcomes during cardiac remodeling. Combined with the established protective role of some FGF ligands and endothelial FGFR signaling in I/R injury, this study supports the development of therapeutic strategies that promote cardiomyocyte FGF signaling after I/R injury.

## Introduction

Despite increased awareness, advances in early recognition, risk prevention, and evidence-based treatments, coronary heart disease (CHD) remains the most common type of heart problem, accounting for ~28% of deaths in the US (1). Furthermore, 51% of patients that present with ST-segment elevation myocardial infarction (STEMI) and survive will die within 5 years (1).

Over the last 30 years, cardiac catheterization and timely reperfusion to attempt to restore blood flow to the ischemic myocardium to limit infarct size is now considered the standard of care. However, restoration of blood flow to hypoperfused myocardium results in additional cardiac damage and accounts for a significant contribution to the final size of the infarct and is referred to as ischemia-reperfusion injury (I/R injury) (2). Various pharmacological and interventional strategies, including ischemic post-conditioning, attempt to minimize adverse outcomes following I/R injury that may result in immediate functional deterioration (i.e., myocardial stunning), ongoing cardiomyocyte damage (i.e., infarction), and remodeling of cardiac tissues (i.e., cardiomyocyte hypertrophy, fibrosis) (3).

Any type of myocardial stress, including hypoxia and ischemia, along with angiotensin II, and adrenergic stimulation, all result in increased expression of Fibroblast Growth Factor 2 (FGF2) (4–9). In addition to FGF2, the adult heart also expresses high levels of FGF16 and lower levels of FGF9 and FGF10 (10–13) FGF16 expression is induced by FGF2 and may function to antagonize FGF2 and prevent cardiac hypertrophy (14–16). Cardiomyocytes express modest levels of FGF receptor 1 (FGFR1) and FGFR4 and lower levels of FGFR2 and FGFR3 (17–22). FGFRs are also expressed in other cardiac cell types including endothelial cells and fibroblasts (23, 24).

In animal models, FGF signaling has proangiogenic properties, is cardioprotective in the acute phase, and promotes cardiac remodeling following I/R injury (6, 25–31). Interestingly, chronic overexpression of a constitutively activated FGFR1 in cardiomyocytes (CM-caFGFR1) resulted in significant myocardial hypertrophy, pathological remodeling (myocyte disarray and fibrosis), reduced cardiomyocyte relaxation and potential long term diastolic heart failure (28). By contrast, mice that lack FGF2 (*Fgf2*^−/−^ mice) show worsened outcomes following I/R injury (25, 32). However, the cell type(s) that receive FGF2 signals are not defined, and cardiomyocytes, interstitial cells, inflammatory cells, and vascular components of the heart are all potential targets of FGF2 signaling. Inactivation of *Fgfr1* and *Fgfr2* in endothelial cells (ECs) and hematopoietic cells by conditionally inactivating them with *Flk1*^*Cre*^ or Tie2-Cre had no acute effect on infarct size at 1 day after the initial I/R injury event, but was necessary for neovascularization in the peri-infarct region 7 days after I/R injury, consistent with a functional reparative role for FGF signaling directly affecting the neoangiogenic response of the EC (23).

Here, we address the requirement for FGFR signaling in the cardiomyocyte. We show that inactivation of *Fgfr1* and *Fgfr2* in myocytes worsens the outcome of I/R injury and thus has a baseline cardioprotective role; however, we found that transient over activation of FGFR1 signaling in myocytes did not provide further benefit following I/R injury.

## Materials and methods

### Mice

Mice were housed in a pathogen-free facility and handled in accordance with standard use protocols, animal welfare regulations, and the *NIH Guide for the Care and Use of Laboratory Animals*. All protocols were approved by the Washington University Animal Studies Committee. To create an inducible, conditional inactivation of *Fgfr1* and *Fgfr2* in cardiomyocytes, a breeding scheme was developed utilizing MHC-rtTA transgenic mice (33), mice with the TetO-Cre allele, and mice with *Fgfr1* and *Fgfr2* flanked by *loxP* sites (*Fgfr1*^*f*/*f*^*; Fgfr2*^*f*/*f*^). MHC-rtTA; TetO-Cre; *Fgfr1*^*f*/*f*^*; Fgfr2*^*f*/*f*^ mice are referred to as CM-DCKO mice. The *ROSA26*^*mT*/*mG*^ reporter allele (34) was also included to assess recombination efficiency and specificity. Controls for these experiments include *Fgfr1*^*f*/*f*^*; Fgfr2*^*f*/*f*^ double flox mice (*DFF*) and MHC-rtTA; TetO-Cre mice with wild type or heterozygous *Fgfrs*. The data presented here includes a combination of both types of controls since there were no differences observed between the different control groups. Mice with an inducible, cardiomyocyte-specific overexpression of a constitutively active FGFR1 transgene (MHC-rtTA; TRE-caFGFR1, referred to as CM-caFGFR1) were generated as previously described (28). All mice were maintained on a mixed C57BL/6J; 129X1 genetic background. CM-DCKO, and appropriate control mice were given doxycycline (DOX) chow from 3 to 7 weeks of age (#S3888, 200 mg/kg doxycycline, Bio-Serv) to inactivate *Fgfr1* and *Fgfr2* in cardiomyocytes and then were placed on standard chow. CM-caFGFR1 mice were induced with a single injection of 100 μl DOX (1 mg/ml saline) 4 h before I/R injury.

### Mouse model of closed-chest cardiac I/R injury

The mouse model of closed-chest, regional cardiac I/R injury was performed in the Mouse Cardiovascular Phenotyping Core at Washington University in St. Louis School of Medicine as previously described (25, 35). Briefly, at 8–10 weeks of age, a loose suture was placed around the left anterior descending artery (LAD) (instrumentation). After 7 days, mice were randomly assigned to the study—baseline (instrumentation only) group, 90 min ischemia + 1 day reperfusion, or 90 min ischemia + 7 days reperfusion. Similar numbers of male and female mice were used in this study and no sex-specific differences in phenotype were noted. The surgeon was blinded to mouse genotype and treatment group for all experiments. The overall mortality for the I/R injury procedure is ~8.4% with all deaths occurring after the instrumentation surgery before the onset of ischemia (25).

### Echocardiography

Mouse echocardiography was performed using a Visual Sonics Vevo2100 High-Resolution *in vivo* Imaging System as previously described (25, 36). Echocardiography analysis of cardiac function and wall motion abnormalities was obtained at baseline, during ischemia, after 1 day of reperfusion, and after seven days of reperfusion. All images were obtained by a single operator with expertise in mouse echocardiography who was blinded to genotype.

### Histology

Assessment of cardiomyocyte cross-sectional area, capillary density, and trichrome assessment of infarct size were performed as previously described (25). *ROSA*^*mTmG*^ reporter gene histology was performed as previously described (23, 34). Rabbit anti FGFR1 (ab63601, Abcam, 1:100 dilution) immunostaining was carried out on 5 μm paraffin sections as previously described (23). After overnight incubation with primary antibody (4°C), sections were incubated with secondary antibody for 1 h at room temperature (Alexa Fluor 488; A11029; Invitrogen; 1:200 dilution). Wheat germ agglutinin (WGA) was used to label cell membranes and identify cardiomyocytes (two colors for WGA were used: FITC-labeled, W1126, Thermo Fisher, 1:100 dilution; CF^®^640R-labeled Conjugate, Biotium, 1:100 dilution). TUNEL staining was performed using the DeadEnd™ Fluorometric System (G3250, Promega) following the manufacturer's instructions. Quantitative RT-PCR was performed and analyzed as previously described (23, 25).

### Statistical analysis

All values are expressed as mean ± standard error of the mean (SEM). Echocardiography data for LV infarct size and ejection fraction were compared using analysis of variance with a Tukey post hoc comparison test. The remaining data were compared using a student's *t*-test. Data with a *p* < 0.05 were considered statistically significant.

## Results

### Mouse model for cardiomyocyte-specific loss of FGFR signaling

**Figure 1:**
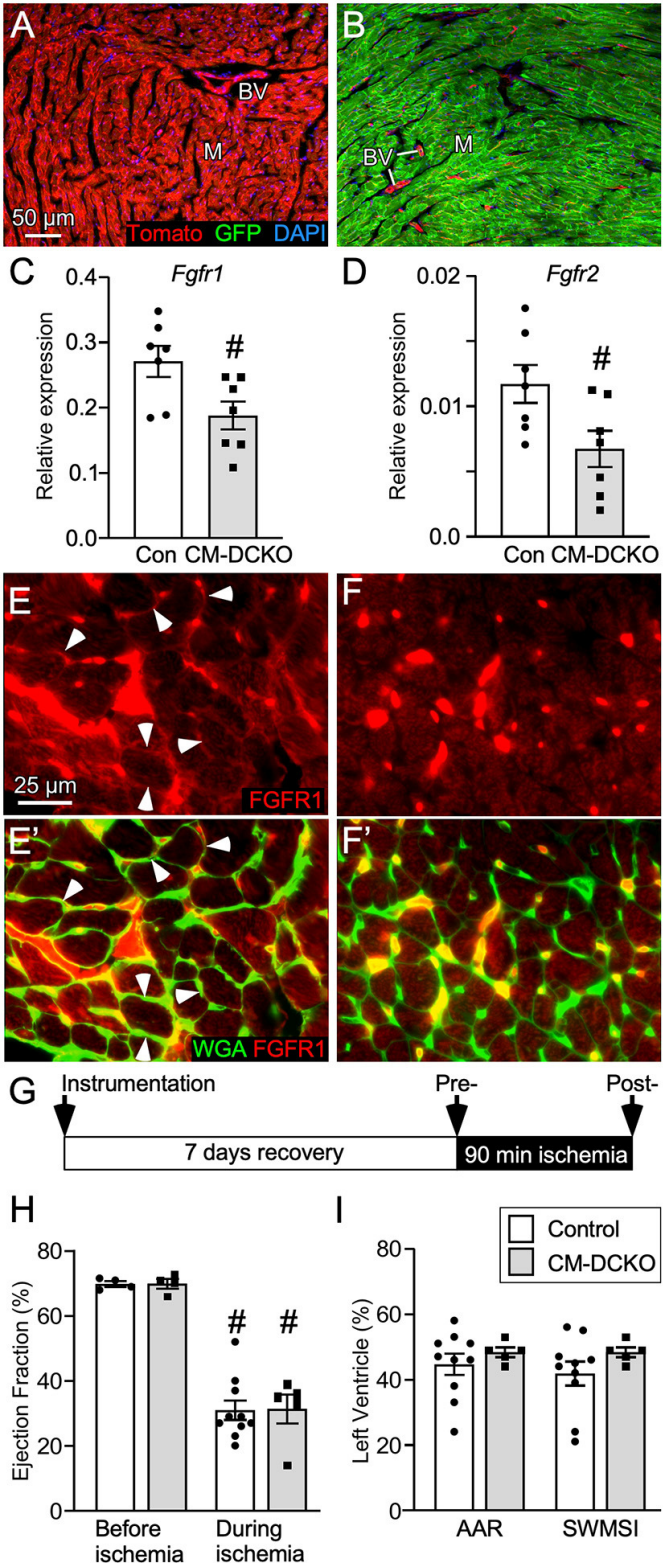
Conditional targeting of *Fgfr1* and *Fgfr2* in adult cardiomyocytes. **(A,B)** Lineage analysis of MHC-rtTA; TetO-Cre; *ROSA*^*mTmG*^ mice. **(A)** In the absence of DOX all cells within the heart express Tomato (red). **(B)** Mice fed DOX chow for 4 weeks beginning at P21 express Cre in myocytes and show recombination in most myocytes (M) which replaces Tomato (red) with GFP (green). Note that blood vessels (BV), endothelial cells, and a few myocytes are still red. DAPI (blue). **(C,D)** Quantitative RT-PCR (TaqMan^®^) of the whole left ventricle from CM-DCKO and DFF control (Con) mice fed DOX chow for 4 weeks showing reduced expression of *Fgfr1* **(C)** and *Fgfr2* **(D)** in CM-DCKO hearts compared to control hearts. # *P* < 0.05 vs control mice. **(E,F)** In control hearts **(E,E')**, immunostaining for FGFR1 (red) shows expression on myocyte membranes (arrowheads) by co-localization with WGA (FITC, green). In CM-DCKO hearts **(F, F')**, no membrane FGFR1 expression was detected. **(G)** Schematic for I/R injury in which a suture is placed around the LAD (instrumentation), mice are allowed to recover for 7d, ischemia is induced by tightening the suture for 90 min and then released to allow reperfusion. **(H)** Measurements of cardiac function before and during ischemia showing significantly decreased ejection fraction during ischemia for both Control (DFF) and CM-DCKO hearts but no significant difference between genotypes (^#^*P* < 0.01 vs. preischemia; *n* = 4–10). (I) Area at risk determined by echocardiography during ischemia, compared to pre-ischemia, showing no difference in the hypokinetic area (AAR) or segmental wall motion score index (SWMSI) between Control and CM-DCKO mice. Scale bar **(A,B)** 50 μm; **(E,F)** 25 μm.

To conditionally target adult cardiomyocytes, MHC-rtTA; TetO-Cre alleles were used to allow doxycycline (DOX)-inducible expression of Cre recombinase in cardiomyocytes. To demonstrate cardiomyocyte-specific targeting, MHC-rtTA; TetO-Cre; *ROSA26*^*mT*/*mG*^ reporter mice were generated. In the absence of DOX, immunofluorescence microscopy demonstrated Tomato expression in all cell types in the heart (Figure 1A). In mice fed DOX chow for 4 weeks, GFP was activated in nearly all cardiomyocytes while Tomato expression (non-targeted cells) remained in vascular endothelial cells, smooth muscle, and a few myocytes (Figure 1B).

To examine the cell-autonomous role of FGFR signaling in cardiomyocytes, cardiomyocyte-specific double conditional knockout mice (CM-DCKO, MHC-rtTA; TetO-Cre; *Fgfr1*^*f*/*f*^; *Fgfr2*^*f*/*f*^) were generated. Controls (Con) included floxed alleles of *Fgfrs* (DFF), and mice lacking either MHC-rtTA or TetO-Cre. All mice were given DOX chow for 4 weeks, beginning at 3 weeks of age. Quantitative RT-PCR of the whole left ventricle showed a relatively high level of *Fgfr1* and a low level of *Fgfr2* expression in control hearts and a 30% and 26% decrease, respectively, in relative expression of *Fgfr1* and *Fgfr2* in CM-DCKO hearts (Figures 1C,D). Immunostaining for FGFR1 identified expression in cardiomyocytes in control hearts (arrowheads in Figures 1E,E') but not in CM-DCKO hearts (Figures 1F,F1'). Baseline hemodynamic values, determined by echocardiography on 8–10-week-old control and CM-DCKO mice showed no significant changes in left ventricle chamber dimensions during systole and diastole, or in systolic ejection function (Table 1).

**Table 1:** Baseline echocardiographic parameters for CM-DCKO mice.

	**Control**	**CM-DCKO**
Heart rate (bpm)	611 ± 16	574 ± 15
Fractional shortening (%)	51 ± 1	51 ± 2
LV mass index	4.1 ± 0.1	4.2 ± 0.3
LV internal diameter (μm) (d)^*^	3.4 ± 0.1	3.5 ± 0.1
LV internal diameter (μm) (s)^*^	1.7 ± 0.1	1.7 ± 0.1
LV post wall thickness (μm) (d)	0.9 ± 0.01	0.9 ± 0.02
LV post wall thickness (μm) (s)	1.5 ± 0.03	1.4 ± 0.06
IV septum thickness (μm) (d)	0.9 ± 0.02	0.9 ± 0.02
IV septum thickness (μm) (s)	1.6 ± 0.04	1.5 ± 0.06

*Data are mean* ± *SEM; n* = *14 control, 7 CM-DCKO*.

* *(d) Diastole, (s) Systole*.

### Mice lacking cardiomyocyte FGFRs have an impaired response to I/R injury

To determine the consequence of inactivation of cardiomyocyte *Fgfrs* during acute cardiac injury and repair, control and CM-DCKO mice were subjected to *in vivo* closed-chest regional cardiac I/R injury (Figure 1G). This model of closed-chest I/R injury permits simultaneous usage of echocardiography during the ischemic period to evaluate immediate effects of LAD occlusion on myocardial wall function. Cardiac function demonstrated significantly decreased ejection fraction during ischemia compared with pre-ischemia values for all genotypes (Figure 1H). Additionally, echocardiographic analysis of wall motion abnormalities (37) showed a similar area at risk (AAR) and segmental wall motion score index (SWMSI) for control and CM-DCKO hearts at the time of ischemia (Figure 1I).

**Figure 2:**
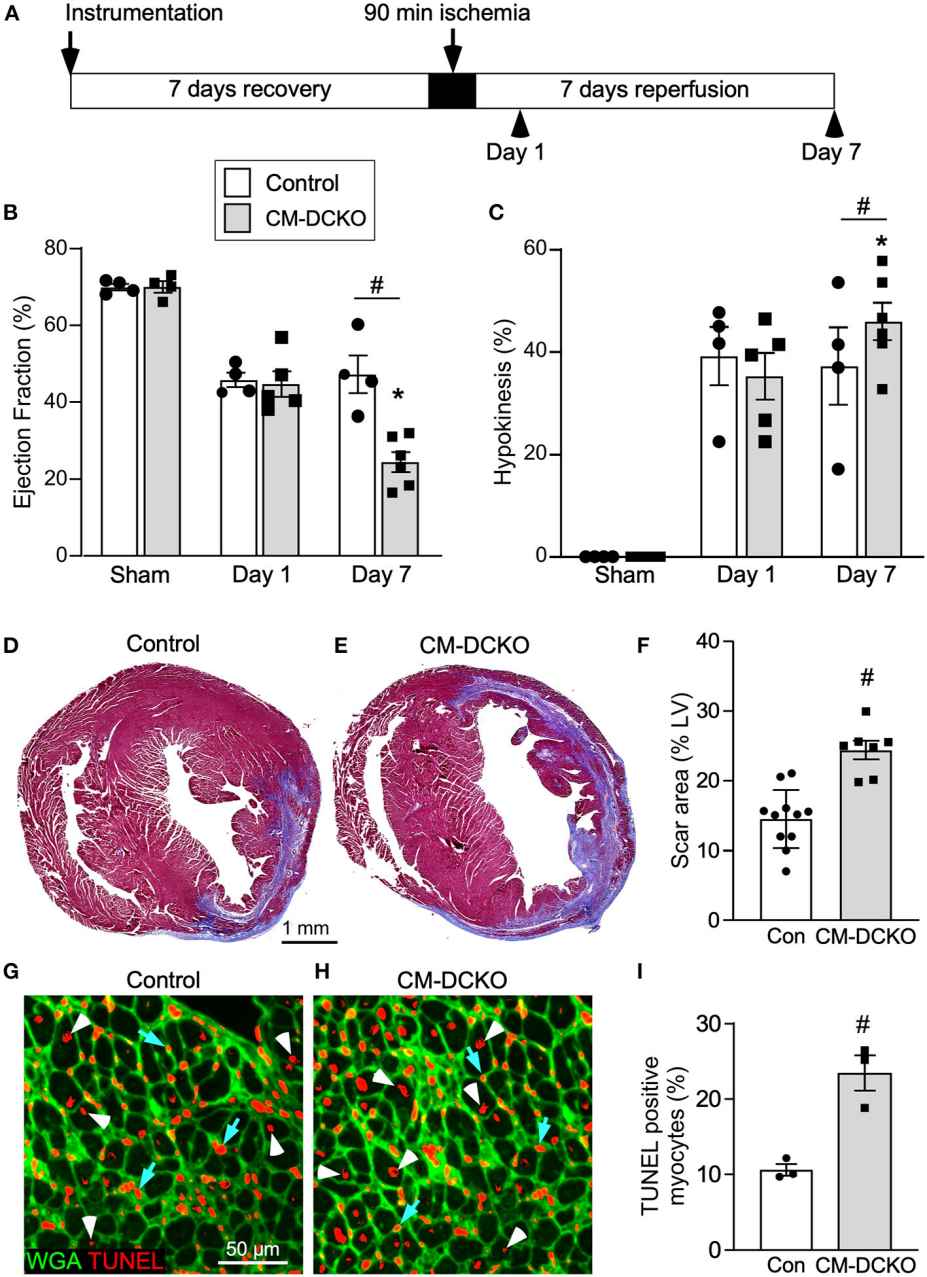
I/R injury model for mice lacking cardiomyocyte *Fgfr1* and *Fgfr2*. **(A)** Schematic showing experimental plan and analysis time points for the acute phase following I/R injury. **(B,C)** Measurements of cardiac function before (Sham) and 1 day and 7 days after reperfusion. Both Control and CM-DCKO mice show a similarly decreased ejection fraction **(B)** and increased hypokinetic area **(C)** 1 day after reperfusion. At 7 days after reperfusion, CM-DCKO mice show a further decrease in ejection fraction and increased hypokinetic area indicating worsened cardiac function. **(D–F)** Histological analysis of scar area (Masson's trichrome stain) 7 days after reperfusion. Relative to Control **(D,F)**, CM-DCKO hearts **(E,F)** show a significantly increased scar area. *n* = 7–11; Scale bar = 1 mm. **(G–I)** Analysis of myocyte cell death in the periinfarct region 1 day after reperfusion showing increased TUNEL positive, WGA (CF^®^640R, green) positive myocytes in CM-DCKO hearts **(H,I)** compared to Control hearts **(G,I)**. TUNEL positive nuclei (red), WGA (green). Scale bar = 50 μm; ^#^*p* < 0.05 vs. control; *p < 0.05 vs. sham.

To characterize genotype-dependent cardiac functional decline after I/R injury we compared echocardiographic changes between control and CM-DCKO mice on day 1 and day 7 after 90 min of *in vivo* closed-chest LAD occlusion (Figure 2A). These time points were chosen to characterize acute effects after IR injury as well as changes occurring during ventricular remodeling (25). Compared to sham operated (instrumented) mice, mice that received I/R injury (both single transgenic controls and CM-DCKO mice) showed a similarly reduced ejection fraction and fractional shortening 1 day after I/R injury. At 7 days after I/R injury, compared to controls, CM-DCKO mice showed a significant further reduction in ejection fraction and fractional shortening (Figure 2B; Supplementary Figure 1A). Volumetric analysis of the LV in both single transgenic controls and CM-DCKO mice that underwent I/R injury demonstrated increased end systolic volume (ESV) 1 day after I/R injury. At 7 days after I/R injury, ESV was further increased in CM-DCKO mice compared to controls (Supplementary Figure 1B). End diastolic volume (EDV) showed a trend to increase by 1 day after I/R injury for all mice and CM-DCKO mice showed a significant increase in EDV compared to sham operated mice at 7 days after I/R injury (Supplementary Figure 1C). Wall motion abnormalities expressed as the hypokinetic part of the LV mirrored the differences in ejection fraction and showed a significantly increased hypokinetic area for all genotypes at 1 day after I/R injury and a further increase in CM-DCKO mice at 7 days after I/R injury (Figure 2C). Qualitative parasternal long and short axis echocardiographic images of control and CM-DCKO mice at 7 days after I/R injury are shown in Supplementary Videos 1, 2, respectively. Quantitative analysis of the infarct area was determined by measuring the fibrotic area of Masson's trichrome stained histological sections at multiple levels through the LV. Qualitatively and quantitatively, CM-DCKO mice demonstrated more extensive infarct area when compared to controls at 7 days after I/R injury (Figures 2D–F).

We hypothesized that inactivation of cardiomyocyte *Fgfrs* could sensitize these cells to ischemia and reperfusion-related stress and result in increased myocyte cell death following I/R injury. To evaluate cell death, histological sections from control and CM-DCKO hearts were stained with WGA to outline myocytes and Terminal deoxynucleotidyl transferase dUTP Nick-End Labeling (TUNEL) assay to assess cell death (38) 1 day after I/R injury. In the peri-infarct region CM-DCKO hearts showed increased TUNEL positive cardiomyocytes compared to control hearts (Figures 2G–I).

**Figure 3:**
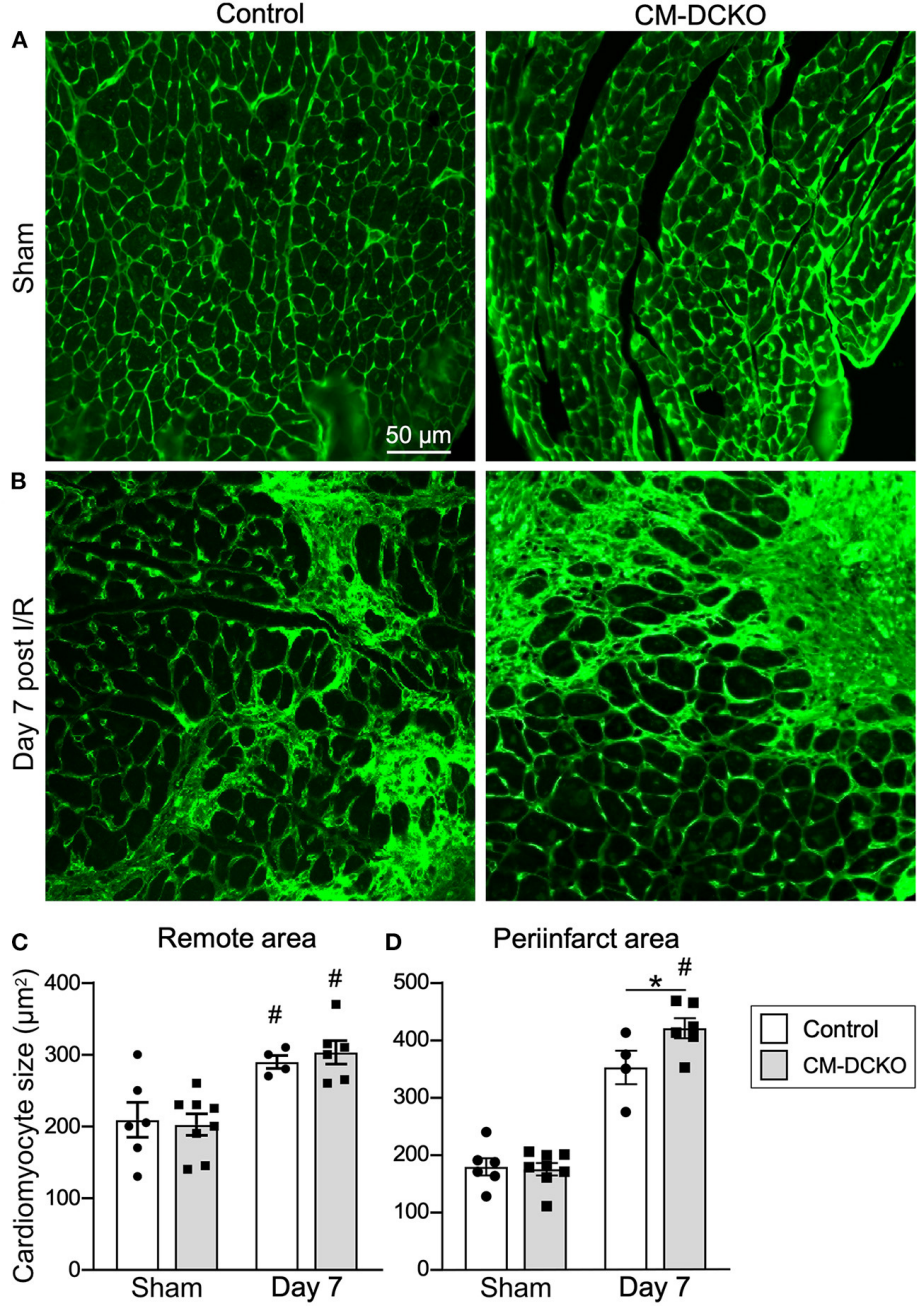
The cardiac hypertrophic response after cardiac I/R injury is minimaly affected by lack of cardiomyocyte *Fgfr1* and *Fgfr2*. **(A,B)** Representative images of WGA (green) stained cardiomyoctyes in the LV free wall of Control and CM-DCKO hearts at 7 days after sham operated (A) or I/R injury **(B)**. **(C,D)** Quantitation of myocyte cross sectional area in the septum [**(C)**, Remote area and **(D)**, periinfarct area] showing no significant difference in the cardiac hypertrophic response between Control and CM-DCKO mice in the remote area and a small increase in myocyte area in the periinfarct region in CM-DCKO mice. Scale bar = 50 μm; *n* = 4–8; ^#^*P* < 0.05 vs. sham (of the same genotype); *p < 0.05 vs. Control.

To estimate myocardial remodeling at the cellular level, WGA-stained histological sections were used to measure cardiomyocyte cross sectional area (Figures 3A,B). Compared to sham operated mice, cardiomyocyte cross-sectional area was significantly increased at 7 days after I/R injury both in the peri-infarct and remote regions in both control and CM-DCKO hearts. At 7 days after I/R injury there was no difference in cardiomyocyte cross-sectional area in control and CM-DCKO hearts in the remote areas (septum); however, in the peri-infarct region, CM-DCKO hearts showed increased cardiomyocyte cross-sectional area compared to control hearts (Figures 3C,D).

**Figure 4:**
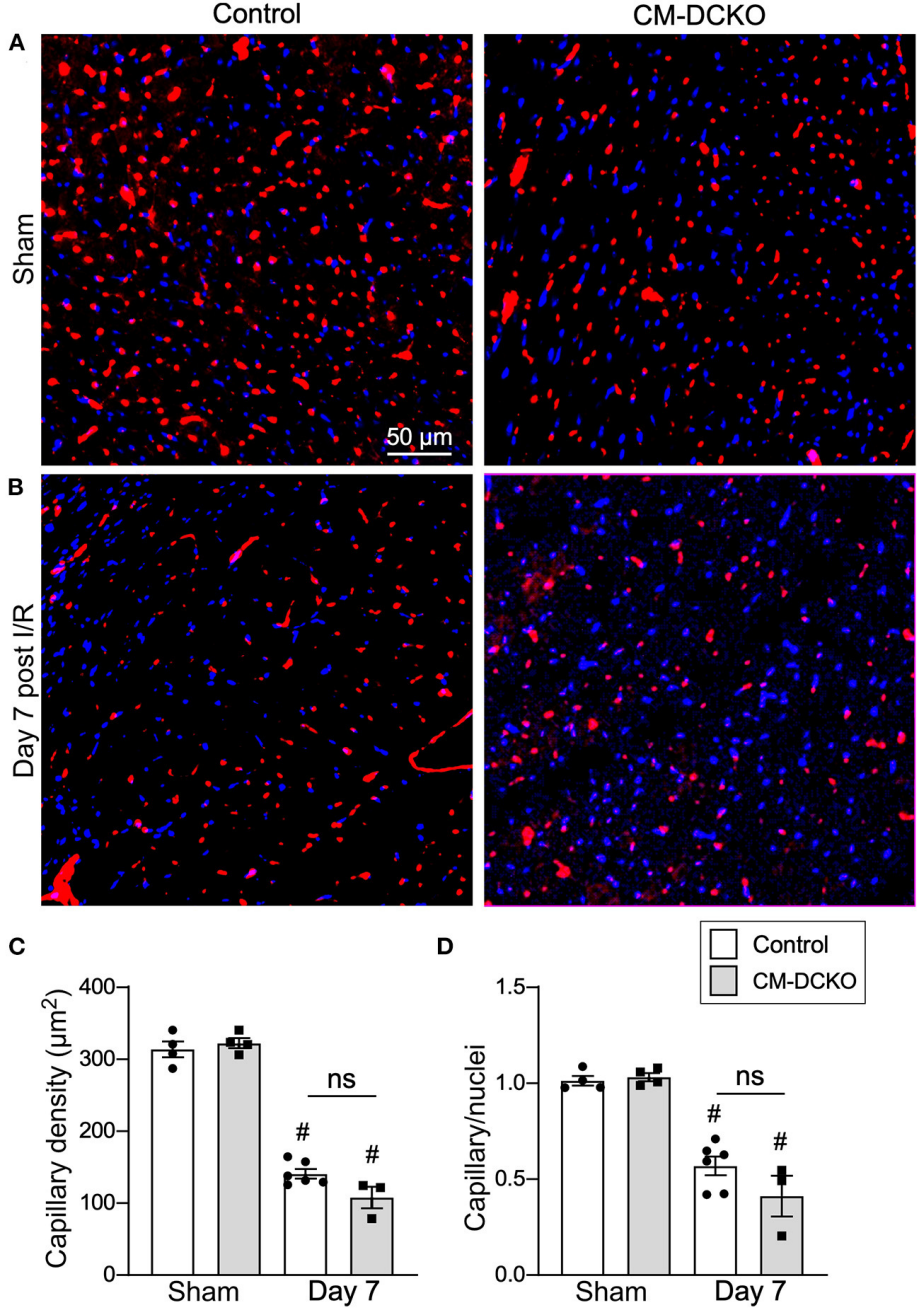
Myocardial capillary density after cardiac I/R injury is not affected by lack of cardiomyocyte *Fgfr1* and *Fgfr2*. **(A,B)** Representative images of immunoflurescence for CD31 (red) and DAPI (blue) showing capillaries in the periinfarct area of Control and CM-DCKO mice 7 days after sham operated **(A)** or I/R injury **(B)**. Scale bar = 50 μm. **(C,D)** Quantitation of capillary density normalized to area **(C)** or to nuclei **(D)** showing a significant capillary rarefaction in the peri-infarct area 7 days after I/R injury without a notable difference in capillary density between Control and CM-DCKO hearts. ^#^*P* < 0.05 vs. sham.

Following I/R injury, the acute vascular rarefaction in the ischemic and peri-ischemic zones leads to compensatory neoangiogenesis. To determine whether loss of cardiomyocyte *Fgfr1* and *Fgfr2* affected vascular and capillary remodeling, capillary density was analyzed in sham opperated hearts and in hearts 7 days after I/R injury (Figures 4A,B). Quantitation of capillary density, normalized to area or number of nuclei showed reduced capillary density and number 7 days after I/R injury but no significant difference between control and CM-DCKO hearts (Figures 4C,D). Myocardium remote to the area of infarction did not show any changes in capillary density compared to sham operated mice (not shown).

### Mouse model for cardiomyocyte-specific activation of FGFR signaling

**Figure 5:**
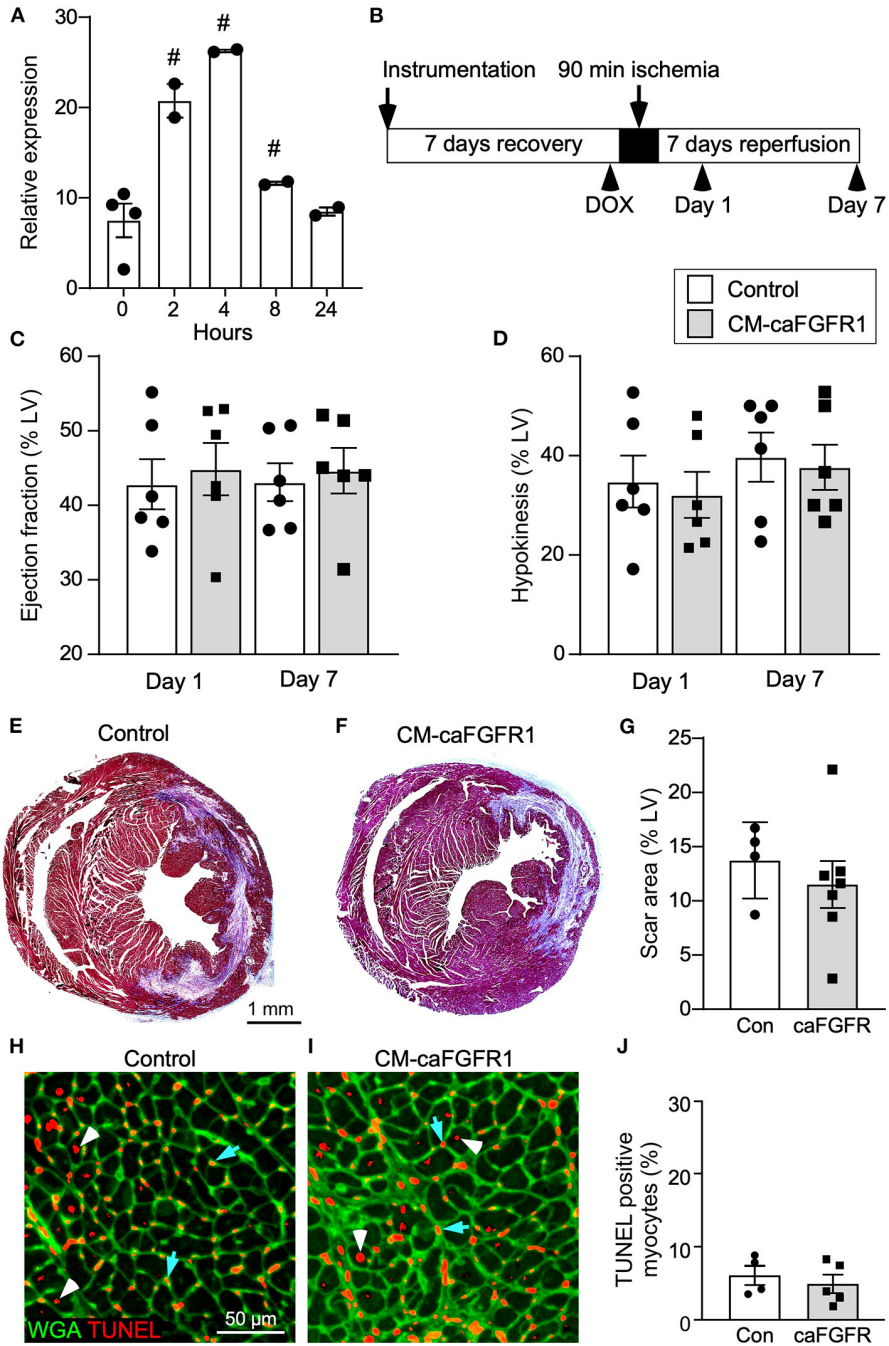
Conditional targeting of an inducible constitutively-active FGFR1 in adult cardiomyocytes. **(A)** Quantitative RT-PCR (TaqMan^®^) of the whole left ventricle from CM-caFGFR1 mice shows rapid induction and peak expression of caFGFR1 4 h after an IP injection of DOX (100 μg). ^#^*p* < 0.05 relative to time 0. **(B)** Schematic showing experimental plan and analysis time points for cardiomyocyte activation of FGFR1. DOX (100 μg) was injected IP 4 h before ischemia. **(C,D)** Compared to sham operated mice (not shown), both Control and CM-caFGFR1 mice show a similarly decreased ejection fraction **(C)** and increased hypokinetic area **(D)** 1 day after reperfusion that was unchanged 7 days after reperfusion. **(E–G)** Histological analysis of scar area (Masson's trichrome stain) 7 days after reperfusion. Relative to Control **(E,G)**, CM-caFGFR1 hearts **(F,G)** show similar scar area. *n* = 4–7; Scale bar = 1 mm. **(H–J)** Analysis of cell death in the periinfarct region 1 day after reperfusion showing similar numbers of TUNEL positive (red), WGA positive (CF^®^640R, green) myocytes in CM-caFGFR1 hearts **(I,J)** compared to Control hearts **(H,J)**. Scale bar = 50 μm.

To determine whether cell autonomous activation of FGF signaling in cardiomyocytes during acute ischemia and reperfusion could further protect the heart from ensuing damage, we utilized a DOX-regulatable TET-on system to transiently induce expression of a constitutively active FGFR1 transgene in cardiomyocytes. MHC-rtTA; TRE-caFGFR1 mice (CM-caFGFR1 mice) and single transgenic controls were induced with a single intraperitoneal (IP) injection of DOX (100 μg) 4 h before the start of ischemia. We found that the 4 h time point corresponded with peak mRNA levels which then returned to baseline by 24 h (Figure 5A).

Echocardiographic analysis of CM-caFGFR1 mice showed similar values at baseline and 4 h after administration of DOX for hemodynamic function, LV systolic ejection function, and dimensions during systole and diastole (except for LV posterior wall during systole that was thinner and carries unkonwn clinical significance) (Table 2). Following DOX administration and I/R injury (Figure 5B), both single transgenic control mice and CM-caFGFR1 mice demonstrated a significant, but similar decrease of their ejection fraction, fractional shortening, ventricular wall motion hypokinesis, and left ventricular volumetric parameters (ESV and EDV) at day 1 and day 7 after I/R injury compared to sham operated mice (Figures 5C,D; Supplementary Figures 2A–C). Qualitative parasternal long and short axis echocardiographic images of control and CM-caFGFR1 mice at 7 days after I/R injury is shown in Supplementary Videos 1, 2, respectively.

**Table 2:** Baseline echocardiographic parameters for CM-caFGFR1 mice.

	**Baseline**	**4 h after induction**
Heart rate (bpm)	591 ± 39	602 ± 44
Fractional shortening (%)	49 ± 2	48 ± 2
LV mass index	108 ± 4	109 ± 4
LV internal diameter (μm) (d)^*^	3.1 ± 0.1	3.1 ± 0.1
LV internal diameter (μm) (s)^*^	1.5 ± 0.1	1.4 ± 0.1
LV post wall thickness (μm) (d)	1.0 ± 0.03	1.0 ± 0.03
LV post wall thickness (μm) (s)	1.5 ± 0.03	1.4 ± 0.03^#^
IV septum thickness (μm) (d)	1.0 ± 0.03	1.1 ± 0.04
IV septum thickness (μm) (s)	1.5 ± 0.03	1.4 ± 0.05

*Data are mean* ± *SEM; n* = *6 control, 6 CM-caFGFR1*.

* *(d) Diastole, (s) Systole*.

# *p* < *0.05*.

**Figure 6:**
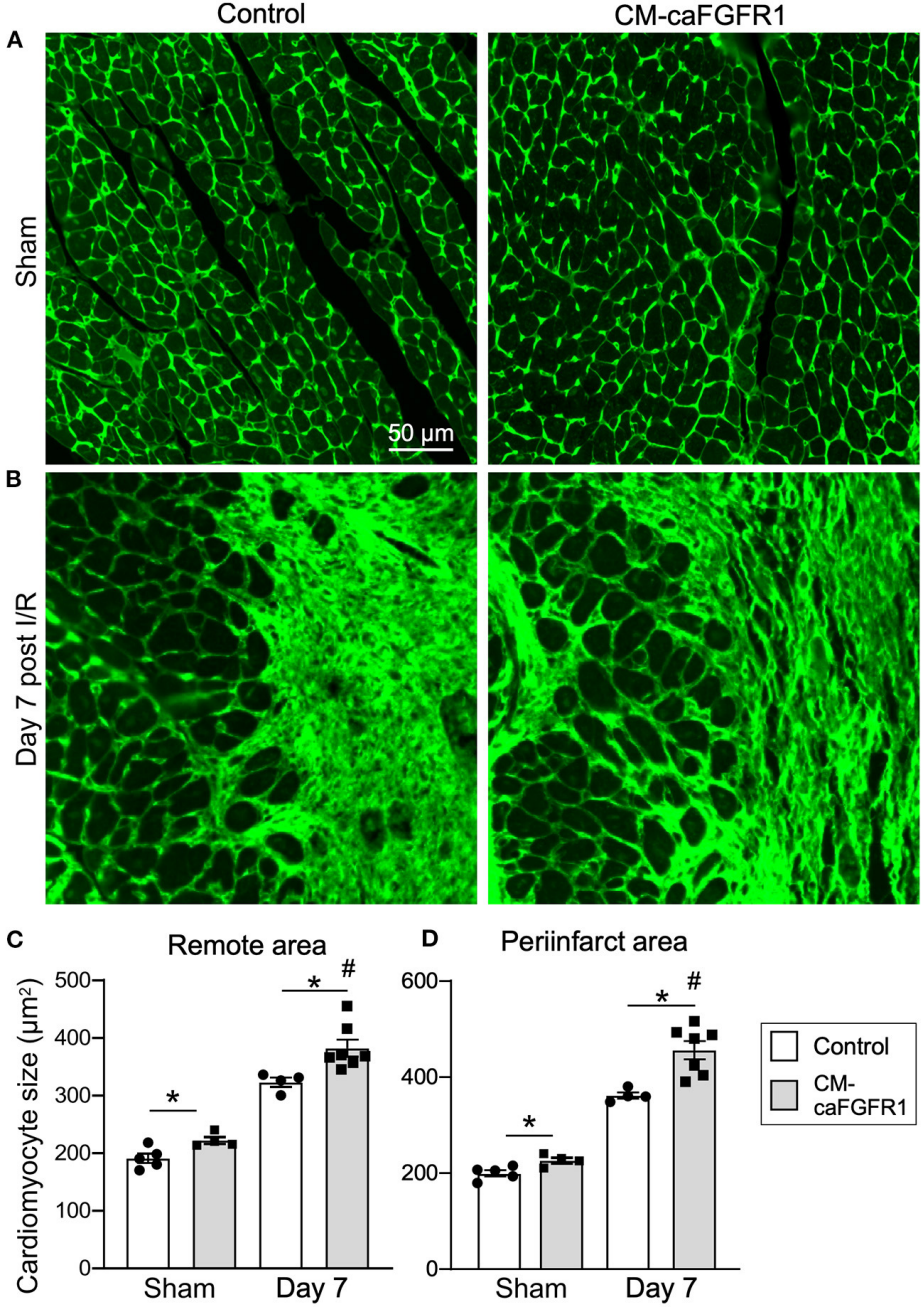
The cardiac hypertrophic response after I/R injury is minimaly affected by transient FGFR1 activation in myocytes. **(A,B)** Representative images of WGA (FITC, green) stained cardiomyoctyes in the LV free wall of Control and CM-caFGFR1 hearts at 7 days after sham operated **(A)** or I/R injury **(B)**. **(C,D)** Quantitation of myocyte cross sectional area in the septum [**(C)**, Remote area and **(D)**, periinfarct area] shows a small increase in the cardiomyocyte area in CM-caFGFR1 hearts at baseline and 7 days after ischemia. Scale bar = 50 μm; *n* = 4–8; ^#^*P* < 0.05 vs. sham (of the same genotype); *p < 0.05 vs. Control.

**Figure 7:**
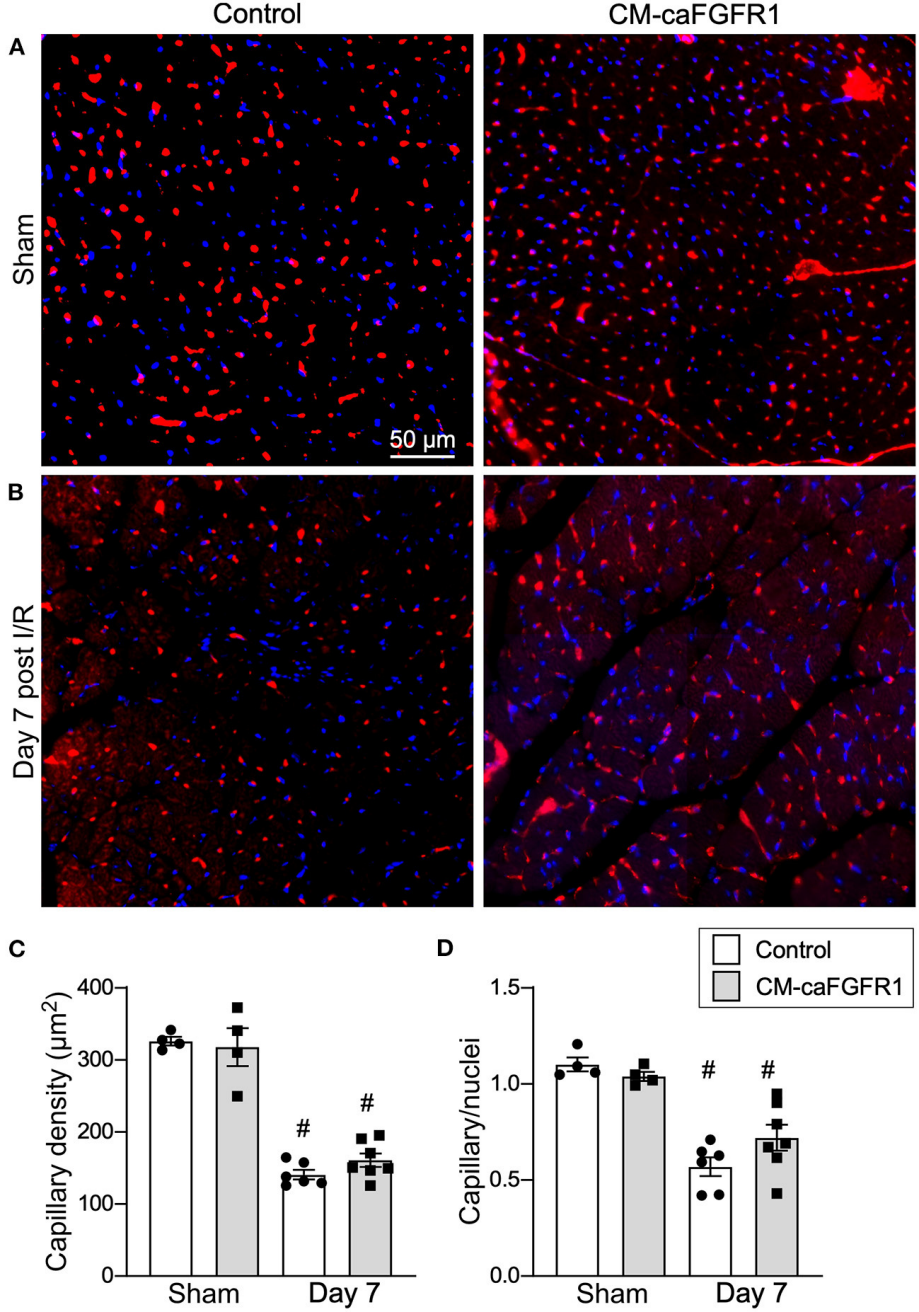
Myocardial capillary density after cardiac I/R injury is not affected by transient FGFR1 activation in myocytes. **(A,B)** Representative images of immunoflurescence for CD31 (red) and DAPI (blue) showing capillaries in the periinfarct area of Control and CM-caFGFR1 mice at 7 days after sham operated **(A)** or I/R injury **(B)**. Scale bar = 50 μm. **(C,D)** Quantitation of capillary density normalized to area **(C)** or to nuclei **(D)** showing a significant capillary rarefaction in the peri-infarct area 7 days after I/R injury without a notable difference in capillary density between Control and CM-caFGFR1 hearts. ^#^*P* < 0.05 vs. sham.

Histological assessement of the LV infarct area was determined by measuring the fibrotic area of Masson's trichrome stained histological sections at multiple levels through the LV. Qualitative and quantitative analysis of control mice and CM-caFGFR1 mice demonstrated a similar scar size 7 days after I/R injury (Figures 5E–G). Analysis of cell death, 1 day after I/R injury, showed similar levels in control mice and CM-caFGFR1 mice (Figures 5H–J). Histological evaluation of cardiomyocyte cross-sectional area showed the expected increase at 7 days after I/R injury in both periinfarct as well as remote areas, and this increase was greater in CM-caFGFR1 mice compared to control mice (Figures 6A–D). Analysis of vascular density and number in the ischemic and peri-ischemic zones showed a similar decrease for both control and CM-caFGFR1 mice (Figures 7A–D).

## Discussion

Myocardial ischemia-reperfusion (I/R) injury elicits multiple adaptive and maladaptive responses that ultimately determine long term outcomes. In mouse models of I/R injury, adaptive responses include compensatory hypertrophy and neoangiogenesis, while maladaptive responses include excessive fibrosis with hypertrophy along with insufficient neoangiogenesis in the periinfarct area (23, 39, 40).

FGF signaling offers one avenue of protection from I/R injury (25, 29, 41–43). Knowledge of the cell autonomous effects of FGF signaling in different cardiac cell types will be critical for optimizing FGF based therapeutic strategies to support cardiac regenerative processes after I/R injury. Consistent with this, we have shown that loss of endothelial FGFR1 and FGFR2 had no effect on vascular development or homeostasis, but did impair cutaneous wound healing, the lung vascular response to hypoxia, and the neovascular response following I/R injury in the heart (23, 30, 44).

FGF signaling is essential for heart development; however, homeostatic roles for FGFs in the heart are poorly defined (13, 41). In the adult heart, we showed that conditional inactivation of cardiomyocyte *Fgfr1* and *Fgfr2* did not affect homeostasis or cardiac physiology; however, following I/R injury these mice showed impaired cardiac function and increased infarct size. Although the contribution of *Fgfr1* and *Fgfr2*, individually, was not investigated, *Fgfr1* is expressed at much higher levels than *Fgfr2*, suggesting that *Fgfr1* would be the dominant receptor with respect to cardiomyocyte survival and physiology following I/R injury. In contrast to the adverse effects of loss of *Fgfr1* and *Fgfr2*, continuously induced expression of a constitutively active FGFR1 in cardiomyocytes resulted in increased contractility within 1 day, and after seven days, hypertrophic cardiomyopathy, left ventricular outflow tract obstruction, diastolic dysfunction and heart failure (28). This supports the idea that FGF signaling could have dose-dependent and time-window dependent protective effects on cardiomyocytes following I/R injury. Here, we compared three different conditions and their outcomes related to cardiomyocyte FGFR activity following I/R injury. We identified a cell-autonomous FGFR signaling requirement for cardiomyocyte survival in the acute phase (1 day) after I/R injury.

Ablation of cardiomyocyte *Fgfr1* and *Fgfr2* or transient activation of FGFR1 in cardiomyocytes did not affect baseline cardiac function or LV volumetric parameters in comparison to littermate controls. Similarly, histological evaluation at baseline before I/R injury, including criteria of myocardial hypertrophy and analysis of vascular remodeling and capillary density did not reveal significant differences between all groups of mice. However, there was a small increase in cardiomyocyte cross-sectional area 7 days after transient expression of caFGFR1 and I/R injury, which is consistent with a previous report (28). Compared to baseline echocardiographic values, echocardiography during LAD occlusion showed no difference in wall motion abnormalities including area at risk, segmental wall motion score index, and ejection fraction for control, CM-DCKO, and CM-caFGFR1 mice.

One day after I/R injury, cardiac function, hypokinesis, and echocardiographic criteria of infarction were similarly affected in all three genotypes/conditions, suggesting that loss of cardiomyocyte FGFR1 and FGFR2 does not adversely affect cardiac function after I/R injury. However, one-day after I/R injury, we did see increased myocyte apoptosis in the LV free wall, which is consistent with previously described protection against cardiac insults provided by FGF ligands (including FGF1, 2, 9, 16, 19, and 21) (25, 42, 45–50). The increased cardiomyocyte death in CM-DCKO mice clearly could contribute to worsened cardiac function, hypokinesis, and echocardiographic and histological criteria of infarction 7 days after I/R injury. Whether the acute increase in cardiomyocyte death results from the initial ischemia or from reperfusion injury cannot be distinguished by these studies. It is also possible that myocyte FGFR signaling could independently support myocyte contractility, as demonstrated in studies that chronically activated myocyte FGFR1 (28). Sustained or enhanced myocyte contractility could improve longer term functional parameters after I/R injury.

Collectively, these data suggest that there is a baseline reserve of FGF signaling capacity in cardiomyocytes that protects cardiomyocytes against the acute effects of I/R injury, and that transiently augmenting this FGF signaling capacity during ischemia and the acute phase of reperfusion is not sufficient to further increase myocyte survival. However, transient activation of myocyte FGFR1 in this study presents some limitations such as the observed higher intra-animal variability with cardiac functional measurements, which could result from variability in the timing or level of transient receptor activation. Future studies could examine whether more sustained or higher-level activation of myocyte FGFR signaling is beneficial.

FGF2 and FGF23 signaling have been shown to promote cardiomyocyte hypertrophy *in vitro* and *in vivo* in pressure overload conditions, while FGF21 had opposite effects (21, 22, 51–55). However, it is not known if this is due to cell-autonomous vs. non-autonomous effects on cardiomyocytes. Interestingly, chronic activation of cardiomyocyte FGFR1 rapidly increased contractility and secondarily increased hypertrophy, a property shared by chronic expression of FGF23 (21, 28). These observations are in agreement with direct measurement of myocyte cross-sectional area that was significantly increased in CM-caFGFR1 animals both in sham and in I/R injury mice seven days after transient expression of CM-caFGFR1 in cardiomyocytes. Interestingly, cardiomyocyte cross-sectional area was also significantly increased in CM-DCKO mice in comparison to control animals, which likely results from compensatory conditioning in response to increased cardiomyocyte loss rather than a direct effect on cardiomyocytes.

We further tested the hypothesis that regulation of cardiomyocyte signaling in control, CM-DCKO, and CM-caFGFR1 mice could indirectly affect the extent of capillary rarefaction and vascular remodeling. In sham operated mice, there was no difference in capillary density among control, CM-DCKO, and CM-caFGFR1 mice in the LV free wall. Although there was a significant reduction in capillary density in the periinfarct region at seven days after I/R injury, there was no significant effect resulting from loss or activation of FGFR signaling in cardiomyocytes. These data suggest that FGFR signaling in cardiomyocytes does not regulate the elaboration of angiogenic factors that maintain adjacent capillary networks or regulate the neoangiogenic response to I/R injury and that cardiomyocyte FGFR signaling may function independently of endothelial FGFR signaling.

In an *ex-vivo* working heart model, a cardioprotective role for FGF2 was mediated by protein kinase C (PKC) α and ε (56). This *ex-vivo* model was further used to show that the 18 kDa form of FGF2 (low molecular weight form) is protective via phosphorylation of phospholamban, a protein that regulates sarco-endoplasmic reticulum calcium ATPase (SERCA) (57). However, the specific FGFR and the intermediary pathway by which FGF2 signaling regulates this phosphorylation event is not known.

Our data suggests that FGFR1, which is expressed in myocytes at much higher levels than FGFR2, is a good candidate receptor to mediate the activities of 18 kDa FGF2. However, cardiomyocytes also express FGFR3 and FGFR4 (21, 43, 58, 59). These receptors could have synergistic or redundant activities with FGFR1 and FGFR2, or potentially antagonistic or independent functions compared to FGFR1 and FGFR2. Future studies will be required to inactivate *Fgfr3* and *Fgfr4*, either alone or in combination with *Fgfr1* and *Fgfr2*. As FGFRs activate multiple downstream signaling pathways (43, 60), future studies will be required to determine *in vivo* which FGFRs control PLCγ/PKC activation and other pathways including MAPK, PI3K/AKT, and Stat in cardiomyocytes.

To determine if brief over-activation of FGFR signaling could add protection, we transiently activated FGFR1 in cardiomyocytes at the time of I/R injury. Although this transient activation did not have a significant protective effect, extending the time course of FGFR1 activation by 1 or 2 days could have more significant protective effects. However, these potential benefits will need to be balanced with potential adverse effects seen with chronic FGFR1 activation (28). We have recently found that different FGFRs can elicit different downstream intracellular signals in the context of lung development (61). Therefore, the downstream signals resulting from induced expression of caFGFR1 could differ from those induced downstream of endogenous FGFRs that are activated by endogenous FGF ligands, such as 18 kDa FGF2.

In conclusion, this study demonstrates that endogenous FGFR1 (and FGFR2) confers cell-autonomous protection to cardiomyocytes that are subjected to I/R injury by reducing cell death and limiting hypertrophy which collectively improves performance outcomes. In the absence of cardiomyocyte FGFR1 (and FGFR2), there is a detrimental effect in response to I/R injury, which leads to increased myocyte death within 1 day following I/R injury and reduced cardiac performance after 7 days.

## Data availability statement

The original contributions presented in the study are included in the article/Supplementary materials, further inquiries can be directed to the corresponding author.

## Ethics statement

The animal study was reviewed and approved by NIH Guide for the Care and Use of Laboratory Animals, Washington University Animal Studies Committee, and Animal Welfare Assurance #D16-00245; Protocol No. 22-0111.

## Author contributions

DM: designed the study, performed experiments, prepared figures, and drafted and reviewed the manuscript. SH: designed the study, performed experiments, prepared figures, and critically reviewed the manuscript. CW and AK: performed experiments and critically reviewed the manuscript. DO: designed the study and critically reviewed the manuscript. All authors contributed to the article and approved the submitted version.

## Funding

Funding for this study was provided by the National Institutes of Health grant HL105732, and funds from the Departments of Developmental Biology and Pediatrics at Washington University School of Medicine.

## Conflict of interest

The authors declare that the research was conducted in the absence of any commercial or financial relationships that could be construed as a potential conflict of interest.

## Publisher's note

All claims expressed in this article are solely those of the authors and do not necessarily represent those of their affiliated organizations, or those of the publisher, the editors and the reviewers. Any product that may be evaluated in this article, or claim that may be made by its manufacturer, is not guaranteed or endorsed by the publisher.

## Supplementary material

Supplemental Figure 1 Echocardiographic measurements of cardiac function before (Sham) and 1 day and 7 days after reperfusion for mice lacking cardiomyocyte *Fgfr1* and *Fgfr2* (CM-DCKO). **(A)** fractional shortening, **(B)** end systolic volume, **(C)** end diastolic volume. ^#^*p* < 0.05 vs. control; ^*^*p* < 0.05 vs. sham.

Supplemental Figure 2 Echocardiographic measurements of cardiac function before (Sham) and 1 day and 7 days after reperfusion for mice activating FGFR1 in cardiomyocytes (CM-caFGFR1). **(A)** fractional shortening, **(B)** end systolic volume, **(C)** end diastolic volume.

Supplemental Video 1 Qualitative depression of systolic function 7 days after I/R injury. Parasternal long axis 2D video clip showing the left ventricle. From left to right: control mouse, CM-DCKO mouse, and mouse with transient activation of FGFR1 in cardiomyocytes.

Supplemental Video 2 Qualitative depression of systolic function 7 days after I/R injury. Parasternal short axis 2D video clip showing the left ventricle. From left to right: control mouse, CM-DCKO mouse, and mouse with transient activation of FGFR1 in cardiomyocytes.
