# Supplementary figures and images for: Fibroblast growth factor receptor signaling in cardiomyocytes is protective in the acute phase following ischemia-reperfusion injury
Source: Front Cardiovasc Med. 2022 Sep 23;9:1011167. doi: 10.3389/fcvm.2022.1011167 (PMC9539275; doi:10.3389/fcvm.2022.1011167)

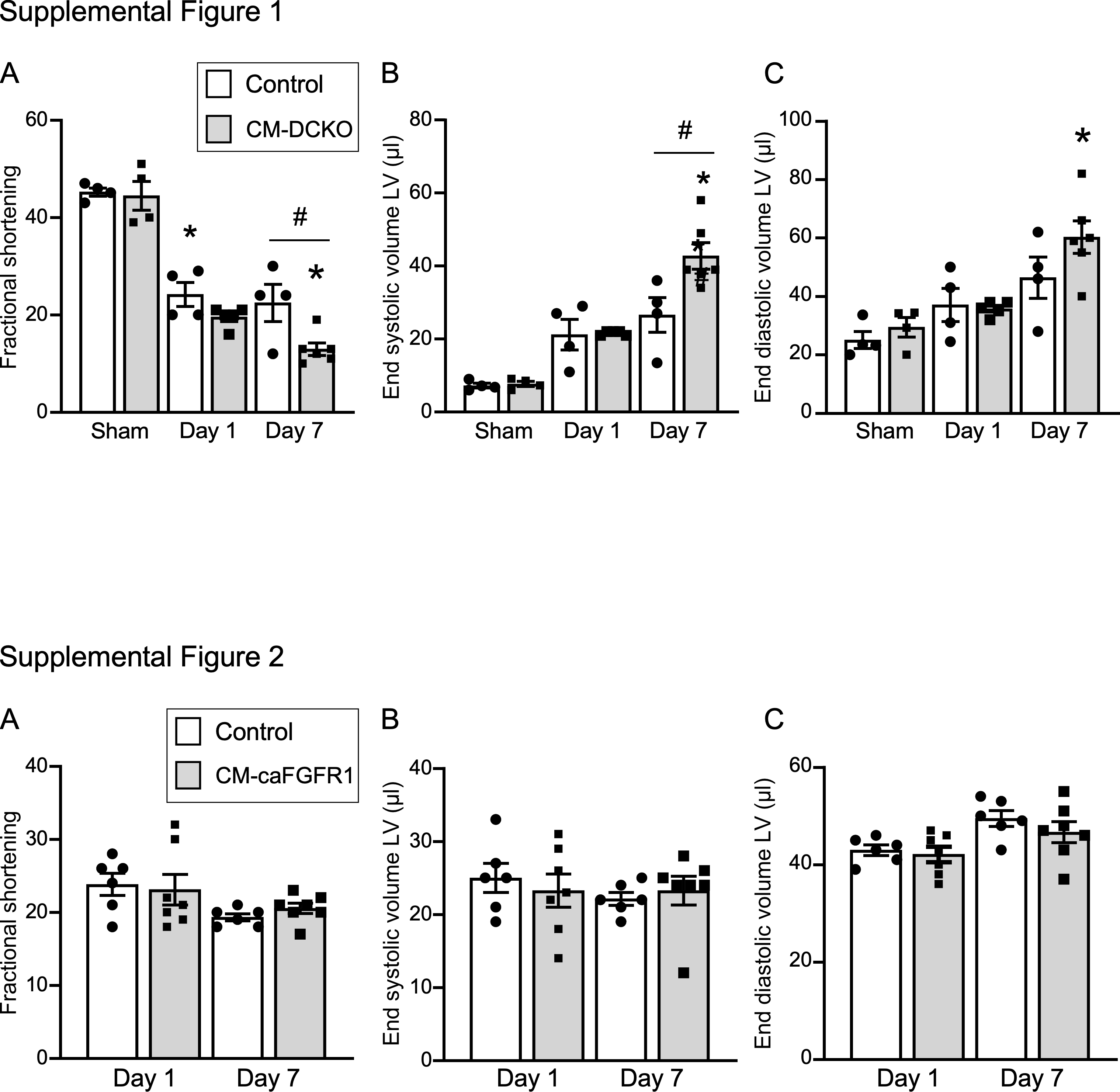

Supplement: Supplemental Figure 1 — Echocardiographic measurements of cardiac function before (Sham) and 1 day and 7 days after reperfusion for mice lacking cardiomyocyte Fgfr1 and Fgfr2 (CM-DCKO). (A) fractional shortening, (B) end systolic volume, (C) end diastolic volume. #p < 0.05 vs. control; *p < 0.05 vs. sham. [file Image_1.JPEG]
